# Robotic Versus Conventional Minimal-Invasive Inguinal Hernia Repair: Study Protocol for a Prospective, Randomized and Blinded Clinical Trial

**DOI:** 10.29337/ijsp.175

**Published:** 2022-06-06

**Authors:** Fiorenzo V. Angehrn, Kerstin J. Neuschütz, Johannes Baur, Romano Schneider, Alexander Wilhelm, Lea Stoll, Julian Süsstrunk, Markus von Flüe, Martin Bolli, Daniel C. Steinemann

**Affiliations:** 1 Clarunis, Department of Visceral Surgery, University Center for Gastrointestinal and Liver Diseases, St. Clara Hospital and University Hospital Basel, Basel, Switzerland

**Keywords:** inguinal hernia, randomized and blinded clinical trial, robotic transabdominal preperitoneal hernia repair (rTAPP), totally extraperitoneal hernia repair (TEP)

## Abstract

**Introduction:**

Inguinal hernia repairs are commonly performed procedures. The surgical techniques vary from open procedures to minimally invasive and robotic-assisted surgeries and include totally extra-peritoneal hernia repairs (TEP) and robotic transabdominal pre-peritoneal hernia repairs (rTAPP). So far, there is no randomized and blinded clinical trial comparing these two surgical approaches. Our objective is to investigate whether rTAPP is associated with a decreased postoperative level of pain.

**Methods:**

This is a prospective, single center, randomized and blinded clinical trial. Patients will receive either rTAPP or TEP for uni- or bilateral inguinal hernias. All patients and assessors of the study are blinded to the randomization. The perioperative setting is standardized, and all surgeons will perform both rTAPP and TEP to eliminate surgeons` bias. Primary endpoint is the assessment of pain while coughing 24 hours after surgery using the numeric rating scale (NRS). Secondary endpoints include the assessment of multiple pain and quality of life questionnaires at several defined times according to the study schedule. Furthermore, intra- and postoperative complications, duration until discharge, procedure time, duration of postoperative sick leave and the recurrence rate will be evaluated.

**Registry:**

The trial has been registered at ClinicalTrials.gov under the registry number NCT05216276.

**Highlights:**

- Trial comparing robotic and conventional minimal-invasive inguinal hernia repair
- Randomized and patient/assessor blinded trial
- Early postoperative pain as primary outcome (24 hours)
- Secondary patient outcomes include pain and quality of life scores up to one year
- Further secondary outcomes: complications, costs, surgeon’s stress level

## 1. Introduction

Inguinal hernias are the most common type of hernias and the reported annual incidence varies from 13 to 34/10,000 [1,2,3]. The only definitive treatment is surgical repair, which is therefore one of the most commonly performed procedures [1].

### 1.1. Surgical procedures for inguinal hernia repair

The applied surgical techniques for inguinal hernia repair have evolved over the past decades [4]. For a long period, open techniques were the gold standard, initially with a suture repair, as in the Bassini or Shouldice technique, later with a tension-free mesh implantation according to the Lichtenstein procedure [5]. Subsequently, minimally invasive techniques such as totally extra-peritoneal hernia repair (TEP) and transabdominal pre-peritoneal hernia repair (TAPP) were introduced [6]. The use of a mesh shows clear benefits regarding recurrence and persisting pain compared to meshless procedures [7]. Furthermore, minimally invasive approaches show a lower rate of acute and chronic pain, faster recovery times and a reduced rate of paresthesia [7,8,9].

Robotic-assisted hernia repair has previously been described as a “natural progression” [10]. Robotic procedures follow the same operative techniques as conventional minimally invasive procedures, yet bring advantages like increased visualization and range of motion [11]. These advantages allow a more meticulous preparation. Both robotic totally extra-peritoneal (rTEP) and robotic transabdominal pre-peritoneal (rTAPP) hernia repair are feasible. Yet, due to technical factors rTAPP is performed more frequently than rTEP [10].

In the literature, comparison of rTAPP and TEP shows controversial results. Some studies report lower pain scores and a lower incidence of complications for rTAPP compared to TEP [12,13]. Others show comparable results regarding postoperative outcomes such as pain, surgical site infection, hospital length of stay and recurrence rate [14,15]. However, so far there is no randomized and blinded study comparing these two techniques and previously mentioned reports show significant differences in preoperative characteristics such as age, body mass index (BMI) and rate of complex cases which may have an impact on postoperative outcomes [15]. Additionally, the further investigation of innovative minimally invasive techniques, like rTAPP, has previously been proposed [14]. With this study we aim to corroborate better evidence in the usage of robotic surgery, especially because the studied procedures belong to the most frequently performed surgeries with and without robotic assistance. To our knowledge, there is no randomized trial investigating the value of robotic surgery in this context. Our goal will be to publish the results in a journal of high impact and therefore supply evidence of high quality that will be incorporated into future guidelines.

### 1.2. Hypothesis

We hypothesize that rTAPP will lead to a reduction of acute postoperative pain compared to TEP and that pain reduction will later translate into earlier recovery. We presume a 20% reduction of the numeric rating scale (NRS) while coughing 24 hours after surgery. Accordingly, the objective of the current randomized controlled trial is to investigate whether rTAPP is associated with a decreased level of pain shortly after surgery compared to conventional TEP.

## 2. Methods

### 2.1. Study design and setting

This is a prospective, single center, randomized and blinded clinical trial. The trial will be led by and conducted at the Clarunis, University Center for Gastrointestinal and Liver Diseases in the St. Clara Hospital in Basel, Switzerland.

### 2.2. Registration

This trial has been registered at ClinicalTrials.gov under the registry number NCT05216276.

### 2.3. Inclusion and exclusion criteria

**Figure 1:**
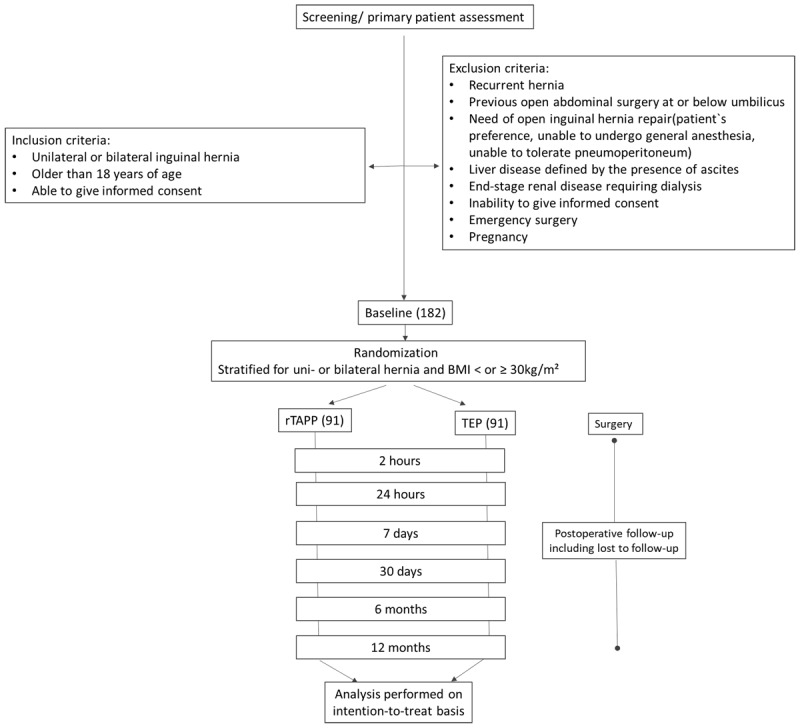
Flowchart of participant recruitment, randomization and follow-up.

Patients with a unilateral or bilateral inguinal hernia, older than 18 years of age and able to give their informed consent are included in the study. Exclusion criteria are recurrent hernias, previous open abdominal surgery at or below the umbilicus, liver disease defined by the presence of ascites, end-stage renal disease requiring dialysis, pregnancy, the inability to give informed consent, and the requirement of emergency surgery. Furthermore, patients with the need of an open surgery, due to their preference, the inability to undergo general anesthesia or to tolerate a pneumoperitoneum will not be included. A flowchart of participant recruitment, randomization and follow-up is shown in Figure 1.

### 2.4. Screening/Primary patient assessment

All patients seen during consultation for inguinal hernia will be registered. This primary assessment will be performed for every patient, including patients that withhold consent or fulfill any of the mentioned exclusion criteria, hereby ensuring a thorough documentation and a complete dataset.

### 2.5. Sample size determination

A numeric rating scale (NRS) of 4.37+/–1.66 at coughing 24 hours after surgery is assumed in the control group. This estimate takes two previous studies into consideration: the double-blinded randomized controlled trial by A. Mughal comparing TEP with and without peripheral nerve block [16] and the retrospective study analysis by C.-C. Yu comparing the mean NRS after TEP with glue versus tack fixation of the mesh [17]. The study hypothesis is a 20% reduction of the NRS. Given a level of significance of 0.05 and a power of 0.90 a sample size of 76 patients per group is needed. With an estimated dropout rate of 20%, we plan to include 91 patients per group and thus a total of 182 patients.

### 2.6. Randomization, allocation, and blinding

Patients are allocated to rTAPP or TEP by randomization. The randomization is performed as a block randomization and stratified for unilateral or bilateral hernias and for the body mass index (smaller or greater than and equal 30 kg/m^2^). This ensures that the groups will be similar regarding these important covariates. The randomization is performed by a case manager that is not involved in the treatment of the patients. The randomization is performed software-based using the REDCap randomization module. All patients and assessors of the study variables are blinded to the randomization. The surgeon is not blinded. The surgeon will not assess information for the endpoints. The exact surgery is not mentioned in the primary report before surgery nor in the surgery protocol given to the patient or to the postop personnel. The exact report is published only after 7 days. In addition, case manager, anesthesia-, OR-, wake-up-, ward- (if hospitalized) personnel are informed about the blinded study. Given the fact that for rTAPP the incisions are made in a horizontal line at the level of the umbilicus and for TEP the incisions are made in a vertical line between the umbilicus and the symphysis, all patients will receive the same standardized opaque wound dressing, covering all potential incision sites. This ensures the blinding of the patients and the assessors of the study variables postoperatively. Patients will be unblinded 7 days after the surgery. Any violation of the blinding will be noted in the case reporting form.

### 2.7. Participating surgeons

All participating surgeons will perform rTAPP as well as TEP. Each participating surgeon must have performed more than 30 rTAPP and more than 30 TEP within the last 12 months. Furthermore, every surgeon must have performed more than 50 advanced robotic procedures before the beginning of the study and have broad experience in minimally invasive abdominal surgery. The cases for the learning curve for rTAPP and TEP are debated in the literature. It depends not only on the procedure but also on the experience of the surgeon overall. With this double number of 50 advanced robotic procedures and 30 TEP’s and 30 rTAPP’s we want to make sure even if the 30 might be in the lower end of the literature that these surgeons have most likely overcome the learning curve because they are overall experienced surgeons. Advanced robotic procedures include: esophagectomy, gastrectomy, fundoplication, colon resection (right, transverse, left, sigmoid), TME, mesh-rectopexy and TARUP. The operation scheduling will equally distribute rTAPP and TEP for each surgeon, to ensure an elimination of surgeons’ bias.

### 2.8. Perioperative setting

Due to Swiss regulations, surgical treatment of a unilateral hernia is performed in an outpatient setting, whereas patients with bilateral hernias are hospitalized for at least one night. All patients will receive an intravenous antibiotic prophylaxis with 2 g of Cefazolin 30 minutes before the incision. The patients will receive a standardized general anesthesia with an endotracheal intubation, and intraoperative pain management will follow a standard based on fentanyl. Postoperatively, all patients will receive Celecoxib 200 mg twice a day for 5 days and Metamizole 500 mg 4 times a day for 7 days.

### 2.9. Surgical techniques

#### 2.9.1. Robotic transabdominal pre-peritoneal hernia repair rTAPP

The Xi da Vinci patient cart is placed on either side of the operating table with integrated table motion. The abdomen is insufflated with carbon dioxide using a Veress needle at Palmer’s point. The first 8 mm robotic port is placed above the umbilicus and the AirSeal® insufflator is connected hereto. After a brief laparoscopy, two further 8 mm robotic ports are placed on each side of the first port, with a distance of 6–8 cm. After placing the patient in a slight Trendelenburg position, the Xi robot is docked. The monopolar scissors are inserted in the right arm, the fenestrated bipolar grasper in the left arm. The center port holds the 30° endoscope. The peritoneum is incised 4–5 cm above the internal ring, from the median umbilical ligament in direction of the anterior superior iliac spine and gradually detached from the transversalis fascia. Medially the space of Retzius is entered and the Cooper ligament is exposed. Laterally the dissection is completed to the psoas muscle. The hernial sac is separated from the spermatic cord in males or the round ligament of the uterus in females and is then reduced. The peritoneum is then further dissected in cephalad direction for at least 5 cm to create adequate space for the mesh. The mesh with a size of at least 12 × 15 cm is positioned to cover the entire myopectineal orifice including the site of direct, indirect, and femoral hernias. The mesh is sutured to the Cooper ligament and in case of a larger medial hernia additionally to the ventral abdominal wall, using an absorbable Vicryl suture. The triangle of pain is meticulously spared. The initially opened peritoneum is readapted with an absorbable 3-0 V-Loc™. In the end, the ports are removed, the pneumoperitoneum is desufflated, and the skin is closed with a resorbable intracutaneous suture.

#### 2.9.2. Totally extra-peritoneal hernia repair (TEP)

The first incision is made at the umbilicus and access to the layer between the rectal muscle and the posterior lamina of the rectus sheath is gained. Here, a dissecting balloon is inserted to create a preperitoneal space under direct camera visualization and then a 12 mm port is placed. Two further 5 mm ports are installed along the median line below the umbilicus. The patient is placed in a slight Trendelenburg position and tilted towards the surgeon. Equivalently to the rTAPP procedure, the hernial sac is separated from the spermatic cord or the round ligament of the uterus and then reduced. The peritoneum is further dissected to create sufficient space for the mesh which is then inserted and positioned. The mesh is secured with glue or, in case of a M3 hernia according to the European Hernia Society (EHS) classification [18], with resorbable tacks. The ports are then removed, and the gas is desufflated. Afterwards, the fascia at the umbilicus is closed with a Vicryl suture and the skin is closed with a resorbable intracutaneous suture.

### 2.10. Endpoints

The primary endpoint is the assessment of pain while coughing 24 hours after surgery, measured on a numeric rating scale (NRS). The endpoint was determined considering that pain after inguinal hernia repair is a major issue as it may compromise the quality of life and acute postoperative pain may be seen as a surrogate for the development of chronic pain [19,20,21].

Secondary endpoints include the analysis of the NRS score 2 hours, 7 and 30 days as well as 6 and 12 months postoperatively and the assessment of the inguinal pain questionnaire (sf-IPQ), the European Quality of Life 5 Dimension 5 Level questionnaire (EQ-5D-5L), the ICEpop CAPability measure for Older people (ICECAP-O), the Short-Form Six-Dimension (SF-6D), the 12-item Short Form Survey (SF-12) and the Carolina Comfort Scale (CCS) at multiple defined times within 1 year after the surgery. Furthermore, secondary endpoints include the evaluation of intra- and postoperative complications, according to the Clavien-Dindo classification and the Comprehensive Complication index (CCI), and the assessment of the recurrence rate 6 and 12 months postoperatively. The procedure time, duration until discharge, amount of intraoperative and postoperative pain medication, costs of the treatment, duration of the sick leave as well as the costs of the sick leave and the type of labor that can be conducted will be evaluated. Additionally, the ergonomics of the surgeons measured by the National Aeronautics and Space Administration Task Load Index (NASA TLX) and the Dutch Musculoskeletal questionnaire will be examined.

### 2.11. Data collection and study schedule

**Figure 2:**
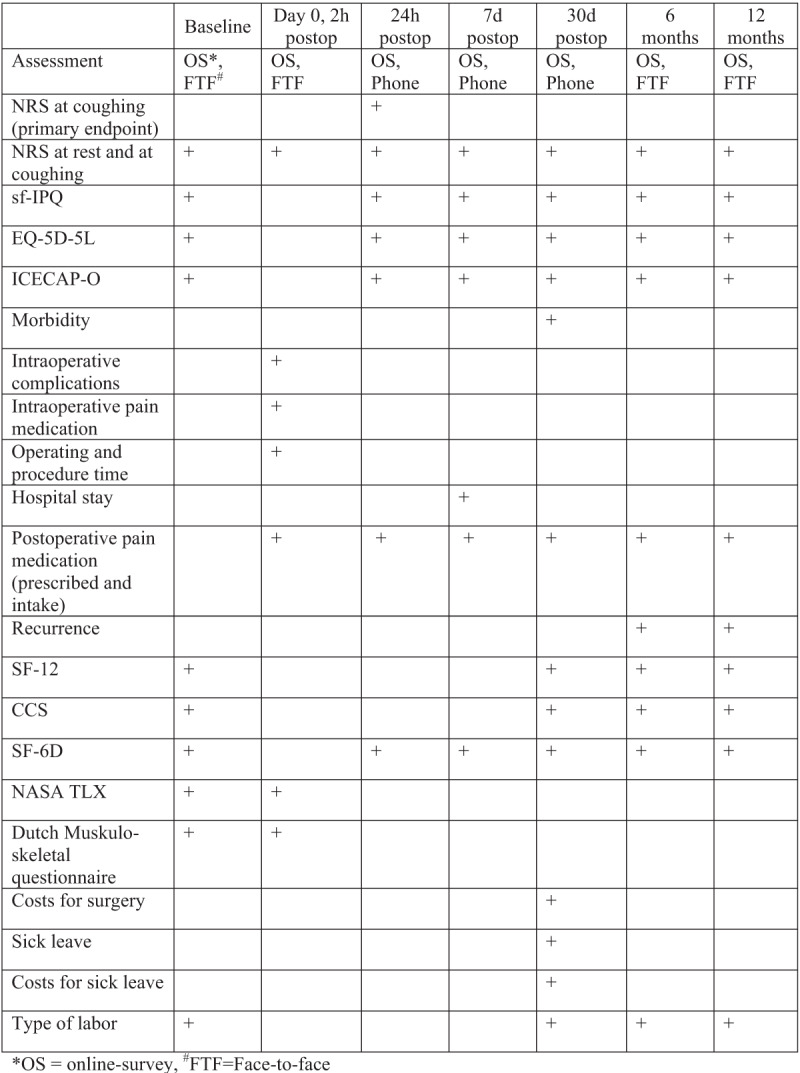
Study schedule including time, manner of assessment and item.

Data is collected by a dedicated study nurse and the operating surgeon face-to-face or via telephone and online survey, after thorough explanation of the different scores and questionnaires according to the schedule displayed in Figure 2. All data is entered in a REDCap study database.

### 2.12. Statistical analysis

Analysis will be performed on an intention-to-treat basis with all patients in the group to which they were allocated. Statistical analyses will be performed using IBM SPSS Statistics software (IBM, Armonk, NY, US), depicting continuous data as mean or median and categorical data as counts and percentage of total. The Mann-Whitney U-test will be used to compare ordinal data and the Fisher’s exact test for the analysis of nominal data. Multiple group comparisons of normally distributed continuous variables will be performed by analysis of variance (ANOVA). Pearson’s r-correlation will be used to correlate the primary and secondary outcomes with surgical experience as well as specific patient characteristics.

## Figures and Tables

**Table:** 

ANOVA	Analysis of variance
BMI	Body mass index
CCI	Comprehensive Complication index
CCS	Carolina Comfort Scale
EHS	European Hernia Society
EQ-5D-5L	European Quality of Life 5 Dimension 5 Level questionnaire
ICECAP-O	ICEpop CAPability measure for Older people
NASA-TLX	National Aeronautics and Space Administration Task Load Index
NRS	Numeric rating scale
rTAPP	Robotic transabdominal pre-peritoneal hernia repair
SF-12	12-item Short Form Survey
SF-6D	Short-Form Six-Dimension
sf-IPQ	Short-form inguinal pain questionnaire
TAPP	Transabdominal pre-peritoneal hernia repair
TEP	Totally extra-peritoneal hernia repair
